# Trials in progress: CARMAN – study protocol of a randomized controlled, international, multicenter, open-label phase II trial evaluating early treatment intensification in patients with high-risk mantle cell lymphoma using CAR-T-cell treatment after an abbreviated induction therapy with rituximab and ibrutinib and 6 months ibrutinib maintenance as compared to standard of care induction and maintenance

**DOI:** 10.1186/s12885-026-16602-1

**Published:** 2026-08-11

**Authors:** Marie-Kristin Tilch, Christian Schmidt, Marek Trneny, Eva Giné, Olivier Hermine, Anke Ohler, Stephanie Herold, Eva Hoster, Linmiao Jiang, Christiane Pott, Martin Dreyling, Georg Hess

**Affiliations:** 1 Department of Hematology and Medical Oncology, University Medical Center of the Johannes Gutenberg University Mainz, Langenbeckstr. 1, Mainz, 55131 Germany; 2 Department of Internal Medicine III, University Hospital, Ludwig Maximilian University (LMU) Munich, LMU Medizin, Marchioninistr. 15, Munich, 81377 Germany; 3 First Department of Internal Medicine -Hematology, First Faculty of Medicine, General University Hospital, Charles University, Prague, Czech Republic; 4 Department of Hematology, Hospital Clínic of Barcelona, IDIBAPS, Barcelona, Spain; 5 CEREMAST Necker Hospital, Paris, France; 6 Institute for Medical Information Processing, Biometry, and Epidemiology (IBE), LMU Medizin, LMU Munich, Munich, Germany; 7 Department of Internal Medicine II, University Hospital Schleswig-Holstein, Campus Kiel, Kiel, Germany

**Keywords:** Mantle Cell Lymphoma, MCL, High risk disease, CAR T-cell therapy

## Abstract

**Background:**

Brexucabtagene-autoleucel (brexu-cel) is an anti-CD19 chimeric antigen receptor T-cell (CAR-T) product approved for relapsed/refractory Mantle Cell Lymphoma (MCL) after two prior treatment lines, including Bruton tyrosine kinase inhibitors (BTKi). Patients with high-risk (hr) disease—defined by high-intermediate or high-risk MIPI-c, p53 overexpression, or TP53 alterations—have a poor prognosis, underscoring the need for improved first-line strategies. The European Mantle Cell Lymphoma Network therefore designed a phase II trial to investigate the incorporation of brexu-cel into first-line therapy for hr MCL.

**Methods:**

CARMAN is a randomized controlled, international, multicenter, open-label phase II trial evaluating efficacy, safety, and tolerability of an abbreviated induction followed by first-line brexu-cel and 6 months Ibrutinib maintenance (Arm A) as compared to standard of care induction and maintenance (Arm B). In Arm A, induction consists of two cycles of ibrutinib plus rituximab (I + R) followed by two cycles of R-CHOP plus ibrutinib (I). R-CHOP + I may be omitted in patients achieving complete or partial remission after two cycles of I + R, who then receive one additional I + R cycle before brexu-cel infusion and I maintenance. Arm B comprises a TRIANGLE-like regimen based on age, fitness, and investigator choice (alternating R-CHOP plus ibrutinib/R-DHAP or IR-bendamustine), followed by IR maintenance. Overall, 150 patients from five European countries are randomized 1:1. The primary endpoint is failure-free survival from randomization, with failure event defined as the earliest of stable disease at the end of induction (Arm B, or Arm A if brexu-cel is not infused) or within 12 weeks from CAR-T-cell infusion (Arm A), disease progression after induction, or death from any cause. Secondary endpoints include efficacy (overall and complete response rates and PET-negative CR rate 6 months from randomization as assessed according to Lugano criteria, molecular remission rate as measured by MRD), safety and tolerability (adverse events graded according to CTCAE), and patient-reported quality of life as measured by the EORTC-QLQ-C30 and EORTC-QLQ-NHL-HG29 questionnaires.

**Discussion:**

When complete, CARMAN will provide important information on efficacy and safety in first-line CAR-T cell therapy in hr MCL and has the potential to prepare a practice changing confirmatory trial for these difficult-to-treat patients. Recruitment is ongoing.

**Trial registration:**

EU clinical trial number: 2022-502405-15-00.

**Supplementary Information:**

The online version contains supplementary material available at 10.1186/s12885-026-16602-1.

## Background and rationale

Mantle cell lymphoma (MCL) is a rare and frequently aggressive subtype of Non-Hodgkin lymphomas (NHL), accounting for 3–10% of adult NHL cases in Western countries, and it is generally considered incurable [1–3]. The median age at diagnosis is 68, and most patients are diagnosed at an advanced stage [4]. While some patients may initially present with a more indolent disease course (e.g., leukemic or non-nodal variants), the majority will require systemic treatment at diagnosis [5–7]. MCL is characterized by the translocation *t(*11;14), which causes Cyclin D1 overexpression, though secondary genetic changes, such as TP53 alterations, commonly emerge as the disease progresses or are even present at diagnosis leading to a highly aggressive disease phenotype [8]. Prognosis is driven by various risk factors, including the MCL International Prognostic Index (MIPI), which incorporates four key clinical prognostic factors: age, performance status, serum LDH level, and leukocyte count [9]. Patients with high-risk MIPI have a significantly lower 5-year overall survival (OS) compared to those with intermediate or low risk features (34% vs. 63% and 83%, respectively) when treated with chemoimmunotherapy [9, 10]. Additionally, high Ki-67 (≥ 30%), high p53 expression (> 50%) or TP53 alterations, and blastic/pleomorphic histology are associated with poor outcomes in newly diagnosed MCL cases [11]. Despite the identification of various risk groups, treatment decisions for newly diagnosed MCL patients are still primarily based on age, stage and performance status, rather than on prognostic scores or biological markers like TP53 mutations and proliferation index.

First-line treatment for MCL generally includes a combination of polychemotherapy and a CD20-targeting antibody. The addition of high-dose Cytarabine to induction chemo-immunotherapy (CIT) before consolidation with high-dose therapy autologous stem cell transplantation (ASCT) has significantly improved response rates and long-term outcome in younger MCL patients, with progression-free survival (PFS) durations of 5 years or more [12–14]. Although with a short follow-up, with the results of the ECOG-ACRIN and TRIANGLE trial including the oral Bruton’s kinase inhibitor (BTKi) Ibrutinib into induction treatment and as maintenance, ASCT can no longer be recommended at a routine basis [15–17]. For older patients, treatment options include R-CHOP (Rituximab, Cyclophosphamide, Doxorubicin, Vincristine, Prednisone), BR (Bendamustine, Rituximab), or VR-CAP (Bortezomib, Rituximab, Cyclophosphamide, Doxorubicin, Prednisone) while targeted agents are expected to change treatment landscape particularly in combination with chemotherapy. Regardless of age, Rituximab maintenance therapy is routinely administered and should be used since it significantly improves OS [18, 19].

Despite high initial response rates, the most patients eventually experience progressive disease (PD). This is particularly evident for patients with TP53 mutations, who often show poor responses to regimens involving Cytarabine, Rituximab, and ASCT. In the Nordic MCL2 and MCL3 trials, TP53-mutated MCL patients had a median overall survival (OS) of only 1.8 years, with 50% relapsing at 1 year, compared to 12.7 years in TP53-unmutated cases [20]. Consequently, there is a high unmet need for an optimized first-line treatment in hr MCL patients.

The SHINE study, which included ibrutinib in combination with chemotherapy, confirmed its benefit as a first-line therapy, demonstrating a significant increase in progression-free survival (PFS) to 80.6 months for BR + Ibrutinib versus 52.9 months for BR + placebo in elderly patients with newly diagnosed MCL [21]. Other clinical trials explored whether the addition of novel BTKis (e.g., BR +/- acalabrutinib, combination recently approved by the EMA following the results of the ECHO trial [22]) or the replacement of chemotherapy with targeted agents like venetoclax (EudraCT-Nr.: 2020-002935-30) can further improve outcomes. Venetoclax, a potent and selective B-cell lymphoma 2 (BCL2) inhibitor, has shown moderate efficacy in BTKi-refractory MCL, with response rates of 40–50% when used alone or in combination with anti-CD20 monoclonal antibodies [23]. However, patients with PD under BTKi generally have poor outcomes [24].

Ibrutinib has significantly advanced the treatment of r/r MCL, showing an overall response rate (ORR) of 68%, with 46% of patients achieving partial response (PR) and 22% reaching complete remission (CR) [25]. Over recent years, Ibrutinib has become standard of care for relapsed patients, however options in the post BTKi-scenario remained scarce [26, 27]. The successful introduction of CAR-T cell therapy has been one of the major advances in the past decade. Anti-CD19 CAR-T cell therapy involves autologous human T cells engineered to express a single-chain variable fragment targeting CD19, linked to intracellular signaling domains from CD28 and CD3ζ molecules. Brexucabtagene autoleucel (brexu-cel), an anti-CD19 CAR-T product, was primarily studied in the ZUMA-2 trial, a multicenter, international, single-arm phase II study involving 68 patients with relapsed MCL after a median of three prior lines of therapy, including BTKi [28], and is FDA and EMA approved for this indication. Patients underwent leukapheresis, lymphodepleting chemotherapy, followed by brexu-cel infusion at a targeted dose of 2 × 10^6 CAR-T cells/kg, based on trials with axicabtagene ciloleucel in refractory aggressive lymphoma [29, 30]. Among the patients, 25% had blastoid histology, 6% had TP53 mutations, and 32% had Ki-67 > 50%. In ZUMA-2, 93% of patients achieved an objective response, including 67% who achieved a complete response. At a median follow-up of 12.3 months, 57% of patients remained in remission. The estimated PFS and OS at 12 months were 61% and 83%, respectively. High objective response rates were also observed in patients with hr features, including a Ki-67 proliferation index ≥ 50%, blastoid or pleomorphic morphology, and TP53 mutations, suggesting that brexu-cel may provide clinical benefit in patient populations typically associated with poor prognosis. Recently, the primary analysis of ZUMA-2, Cohort 3, demonstrated a high ORR (91%) and CR rate (73%) in patients with r/r MCL who were naive to BTKi, consistent with Cohort 1 of ZUMA-2 (BTKi-exposed patients) [31]. While the trial lacked a comparator arm, a recent real-world analysis comparing post-BTK failure treatments with standard therapies confirmed the favorable outcomes of CAR-T therapy [32].

Similar results were observed with lisocabtagene maraleucel (liso-cel), reinforcing the therapeutic potential of CAR-T therapy in r/r MCL, with an ORR of 84% and 66% of the patients achieving CR [33]. However, long-term follow-up is needed to assess the durability of remissions. Compared to allogeneic stem cell transplantation, CAR-T products offer potential major advantages, such as reduced morbidity and mortality, preserved T-cell function, and lower infection risks. However, it remains unclear whether earlier use of CAR-T cell therapy alone or in combination with BTKis can reduce relapse rates in hr MCL patients.

The Australian phase II TARMAC study evaluated the combination of time-limited Ibrutinib (BTKi priming strategy before leukapheresis, with continuous BTKi treatment during conditioning and for 6 months after CAR-T-cell infusion) and the commercially available CAR-T-cell product tisagenlecleucel (tisa-cel) in 20 patients with r/r MCL [34]. The median number of prior treatment lines was 2, with 50% of patients having previously been treated with a BTKi. The primary endpoint (CR rate at 4 months post-infusion) was achieved, with 80% of patients showing CR, and 70% and 40% of patients showing negative measurable residual disease (MRD) by flow cytometry and molecular methods, respectively. At a median follow-up of 13 months, the estimated 12-month PFS rate was 75%, and OS was 100%. Efficacy was maintained regardless of prior BTKi exposure or TP53 mutation status. These results support the potential of combining BTKi with CAR-T-cell therapy, highlighting the safety of post-CAR-T infusion BTKi treatment.

Given that T-cell fitness, which is critical for the efficacy of brexu-cel, deteriorates following prior immunochemotherapy [35], administering brexu-cel as part of first-line treatment without preceding chemotherapy may represent an especially effective strategy to optimize the full potential of CAR-T cell therapy. In patients with chronic lymphocytic leukemia (CLL), Ibrutinib enhanced the in vivo persistence of activated T cells, reduced the Treg/CD4 + T cell ratio, and lowered the immune-suppressive effects of CLL cells through both BTK-dependent and -independent mechanisms [36]. Furthermore, Ibrutinib has been shown to reverse the exhausted T-cell phenotype by decreasing the expression of PD1 and cytotoxic T-lymphocyte-associated protein 4 (CTLA-4) probably through ITK inhibition. Notably, CAR-T-cell generation in the presence of Ibrutinib resulted in improved T-cell viability, enhanced CAR-T-cell expansion, and reduced expression of exhaustion markers such as PD-1, TIM-3, and LAG-3 [37]. Importantly, a second CAR-T-cell treatment in r/r NHL patients showed improved efficacy when Ibrutinib was administered prior to and during the anti-CD19 CAR-T-cell therapy [38], suggesting that Ibrutinib may have synergistic effects with CAR-T cells by enhancing T-cell expansion and preventing exhaustion. While CAR-T-cell therapy may offer curative potential compared to current immunochemotherapy-based approaches, indefinite use of BTKi seems neither justified nor necessary in first-line treatment. Furthermore, recent trial results also demonstrate the efficacy of chemotherapy-free induction, even in hr patients, with a BTKi-based treatment [39–41]. In summary, a BTKi-based induction prior to leukapheresis and CAR-T-cell therapy is justified and reasonable in terms of efficacy, safety and enhanced CAR-T-cell function.

The phase II clinical trial described herein will compare the efficacy, safety, and tolerability of first-line brexu-cel treatment after a shortened induction with R + I versus conventional ICT and Ibrutinib.

### Risk benefit assessment

Ibrutinib is commonly used in CLL and MCL. In patients receiving continuous Ibrutinib monotherapy, most adverse events (AEs) are grade 1 or 2. Common non-hematological AEs include diarrhea (50%), fatigue (41%), peripheral edema (28%), dyspnea (27%), constipation (25%), upper respiratory tract infections (23%), vomiting (23%), and decreased appetite (21%). Pneumonia is the most frequent grade 3–5 infection. Grade 3–4 hematologic AEs include neutropenia (16%), thrombocytopenia (11%), and anemia (10%). Severe bleeding events (grade 3) occurred in 5 of 111 patients, with no grade 4 or 5 events [25]. Cardiovascular risks, particularly atrial fibrillation, are also increased in Ibrutinib-treated patients. Pharmacological interactions between Ibrutinib and P-glycoprotein substrates (e.g., digoxin, dabigatran), CYP3A4 inhibitors and inducers, anti-arrhythmic drugs (e.g., verapamil, amiodarone), direct oral anticoagulants (e.g., apixaban, rivaroxaban), antimicrobials (e.g., azoles, macrolides, rifampicin, carbamazepine), and antiepileptic drugs must be carefully managed [42].

Brexu-cel treatment carries potential risks, predominantly hematological and infectious AEs. In the ZUMA-2 trial, 99% of patients experienced grade 3 AEs, with 94% having cytopenias (neutropenia in 85%, thrombocytopenia in 51%, and anemia in 50%) and 32% developing infections. One-third of patients had grade 3 or higher cytopenias lasting over 90 days post-infusion. Cytokine release syndrome (CRS) and neurotoxicity are commonly seen after CAR T-cell therapy. CRS was observed in 91% of ZUMA-2 patients, mostly grade 1–2, with 15% grade 3 or higher. The interleukin-6 (IL-6) receptor antagonist Tocilizumab, glucocorticoids, and vasopressors were used in 59%, 22%, and 16% of patients, respectively, with no fatalities due to CRS. Neurologic events occurred in 63% of patients, 31% being grade 3 or higher, with 26% receiving tocilizumab and 38% glucocorticoids.

Despite temporary impairments in health-related quality of life (HRQoL) reported 4 weeks after CAR-T therapy in the ZUMA-2 trial, scores improved by 3 months, and some patients reported better HRQoL than before treatment at 6 months [28]. The CARMAN study is expected to show similar benefits. This study will closely monitor patients for CRS and neurotoxicities using validated grading systems like the CRS Grading per Lee [43] and the Immune Effector Cell-associated Encephalopathy (ICE) score. Management includes symptomatic therapies (fluids, antipyretics, vasopressors), tocilizumab, or corticosteroids if necessary. Ibrutinib-related AEs will prompt a dose reduction or treatment hold, and patients will be closely monitored for other toxicities to enable early identification of emerging safety risks. An independent Data Safety Monitoring Committee will review the data to minimize risk.

ZUMA-2 demonstrated surprising efficacy in heavily pre-treated high-risk MCL patients [28]. We expect similar safety results for CARMAN, with potentially improved efficacy as patients will be treatment-naive, preserving T-cell fitness. However, the effect of Ibrutinib pre-treatment still needs evaluation. Given the poor prognosis of hr MCL, the promising efficacy of brexu-cel in earlier trials, manageable toxicities, and the absence of therapy-emergent deaths suggest that the benefits outweigh the risks. Ibrutinib, already approved for r/r MCL, is well tolerated with manageable side effects, and its potential additional efficacy in hr patients makes its use in combination with CAR-T-cell therapy justifiable.

## Methods/study design 

This study is a randomized controlled, international, multicenter, open-label phase II trial evaluating the safety and efficacy of brexu-cel following an abbreviated induction and 6 months Ibrutinib maintenance (Arm A) as compared to standard of care induction and maintenance plus Ibrutinib (Arm B). Study treatment will be administered only to eligible subjects according to inclusion and exclusion criteria after registration and following the randomization result. Arm A consists of 2 cycles of Ibrutinib 560 mg D1-28 p.o. and Rituximab 375 mg/m² i.v. on day 0 or 1 (I + R) and 2 cycles of Ibrutinib 560 mg D1-21 p.o. + R-CHOP (Rituximab 375 mg/m² i.v. on day 0 or 1, Cyclophosphamide 750 mg/m² D1 i.v., Vincristine 1.4 mg/m² D1 i.v., Doxorubicine 50 mg/m² D1 i.v., Predniso(lo)ne 100 mg/d D1-5 i.v. or oral) for primary tumor reduction. In case of good clinical response (CR or PR) after 2 cycles of I + R, Ibrutinib and R-CHOP can be omitted. In this case, one additional cycle of I + R will be applied. In all patients, Leukapheresis will be performed after cycle 2 of I + R, followed by lymphodepleting chemotherapy with Fludarabine (30 mg/m2 D -5 to D -3 i.v.) and Cyclophosphamide (500 mg/ m2 D -5 to D -3) within 14 days after reconstitution after cycle 4 or 3 (Ibrutinib + Rituximab in responders). Brexu-cel will be administered as a single infusion of 2 × 10^6^ anti-CD 19 CAR-T-cells/kg i.v. on Day 0. Response assessments will be performed via imaging and MRD analysis during induction, including a mandatory PET CT scan three months after CAR T cell infusion. Maintenance therapy will be applied with ibrutinib 560 mg/d p.o. for 6 months starting three months after CAR T cell infusion.

In Arm B, standard of care chemoimmunotherapy according to the European standard of care will be applied. Based on the investigator’s choice and the patients’ age and performance status, younger patients (< 65 years or up to 70 years, if considered suitable for intensive regimens) will receive 6 cycles of chemoimmunotherapy: 3 x R-CHOP + I, alternating with 3 x R-DHAP (Rituximab 375 mg/m² D0 or D1 i.v., Dexamethasone 40mgD1-4 oral or i.v., Cytarabine 2000 mg/m² D2 twice daily over 3 h, Cisplatin 100 mg/m² D1 continuously for 24 h (alternatively Oxaliplatin 130 mg/m² D1 i.v.). G-CSF (5 µg/kg) is mandatory in patients receiving R-DHAP from D6 daily until recovery of the white blood count (WBC) > 2.5 G/l Alternatively, pegfilgrastim/lipegfilgrastim may be applied once at D6. Stem cell apheresis may be performed after the second or third cycle R-DHAP according to local standards if the investigator decides to continue with ASCT. Based on the results of the TRIANGLE study, which demonstrated that ASCT provides no additional benefit (16), the initial protocol with mandatory ASCT was accordingly amended in 2025, and ASCT is now optional for younger patients. In elderly (≥ 65 years) or frail patients, 6 cycles of R-CHOP + I for 21 days-cycles or Bendamustine 90 mg/m² i.v. on D 1 + 2, Rituximab on D 0 or 1 and Ibrutinib 560 mg p.o. D1-28 for 28 days-cycles will be applied. Ibrutinib maintenance (560 mg/d) will be administered for 2 years. Rituximab maintenance may be added for 3 years depending on national guidelines.

**Fig. 1:**
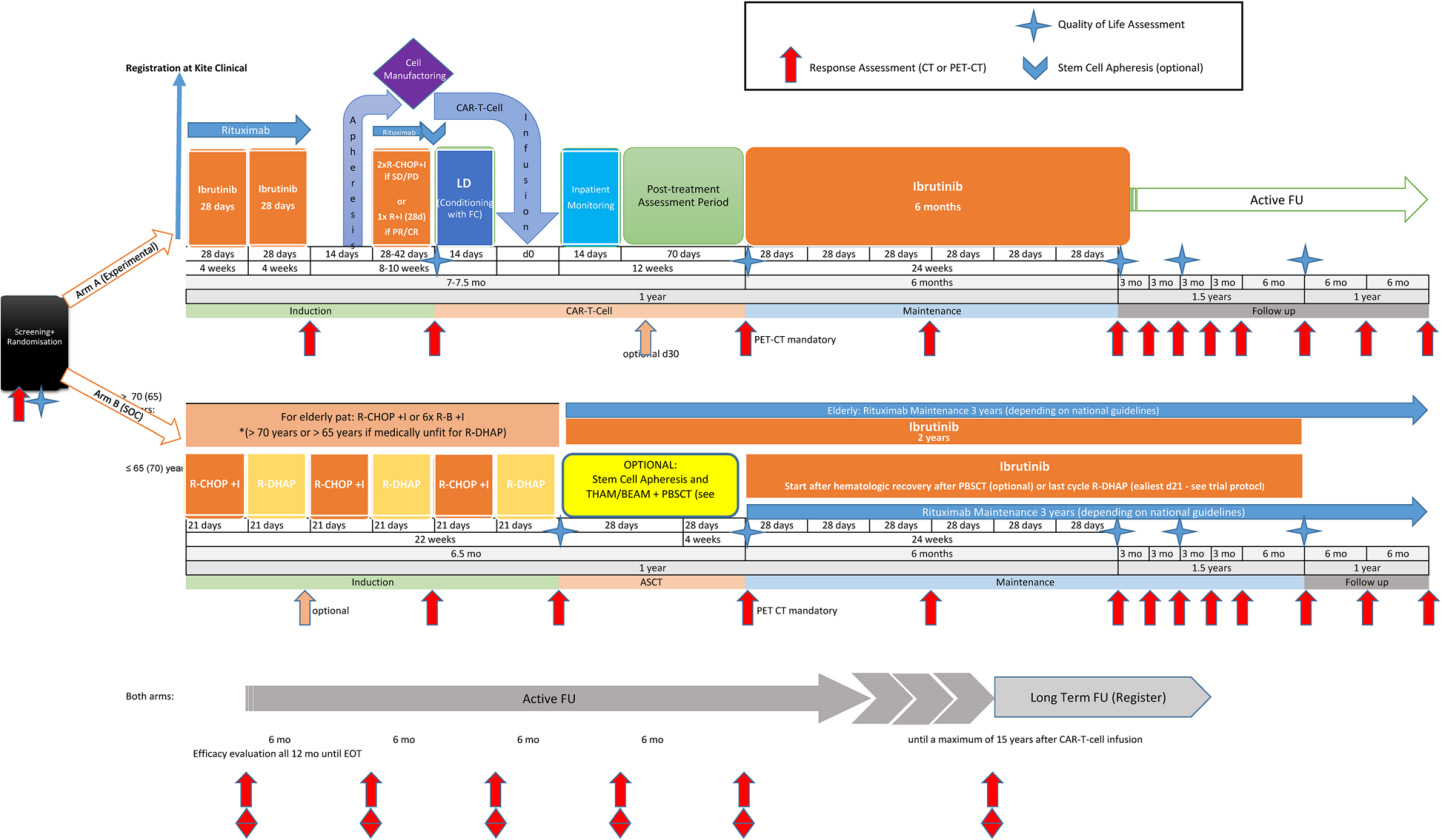
Study design of the CARMAN trial

Figure 1 gives more details on the study design.

The maximal duration of the study will be 7.5 years; with up to 3 years recruitment, 2.5 years of treatment (Arm B) and at least 2 years additional follow-up (FU). Long term FU will be performed outside this study in the European MCL registry (Arm B).

Overall, 40 sites across five European countries are participating (Germany, Spain, France, Czech Republic, The Netherlands) in this investigator-initiated academic study sponsored by LMU Munich. A list of participating study sites can be obtained via e-mail (studyce@med.uni-muenchen.de).

### Study procedures 

Each subject will proceed through the following study periods: screening, registration, randomization, treatment (Arm A or Arm B), active FU and long-term FU. A 1:1 randomization stratified by study group and MIPI risk group (high vs. intermediate/low risk) will be performed centrally via the eCRF by authorized investigators. All treatments will be given open label because it is not feasible to apply all treatment components in a blinded way. Treatments with R-CHOP, R-DHAP and BR are considered standard of care in MCL patients and will be administered according to the standard preparation and infusion procedures of each investigational site. Measures of drug safety and pharmacovigilance will be performed by the investigators and the sponsor.

### Study objectives and endpoints 

The primary objective is to exploratively compare the efficacy of a CAR-T-cell treatment strategy with brexu-cel in previously untreated hr MCL patients (experimental Arm A) versus standard-of-care therapy (control Arm B). The primary endpoint is failure-free survival (FFS) from randomization. A failure event is defined as the earliest of stable disease at the end of induction (Arm B, or Arm A if brexu-cel is not infused) or within 12 weeks from CAR-T-cell infusion (Arm A), disease progression after induction, or death from any cause. Disease response will be assessed according to Lugano criteria.

Secondary objectives are to evaluate the efficacy, safety, tolerability, and patient-reported quality of life associated with the CAR-T-cell treatment strategy. Secondary and exploratory endpoints include overall and complete response rates, progression-free and overall survival, PET-negative complete response at six months from randomization, and molecular remission as measured by minimal residual disease (MRD) after induction and during maintenance in both arms. Safety and tolerability will be assessed according to the Common Terminology Criteria for Adverse Events (CTCAE), and quality of life will be evaluated using the EORTC QLQ-C30 and EORTC QLQ-NHL-HG29 questionnaires at predefined time points.

#### Patients – eligibility

 A total number of 150 patients will be enrolled (randomized 1:1 to each arm), who have to meet the following inclusion criteria: histologically confirmed diagnosis of MCL as per WHO classification, with either cyclin D1 overexpression or *t*(11;14). Participants must present with at least one hr MCL feature, defined as either of high risk MIPI, intermediate MIPI risk with Ki-67 ≥ 30%, TP53 mutation, or p53 overexpression (> 50%). Only treatment-naïve patients aged 18–75 years, with Ann Arbor stage II-IV MCL, are eligible. A measurable lesion is required, with staging including mandatory bone marrow (BM) aspiration and biopsy if BM is involved. The ECOG performance status must be ≤ 2, and specific laboratory values must meet defined thresholds. Participants must have no involvement of the central nervous system, provide written informed consent, use safe contraception methods (for fertile patients), and have a negative pregnancy test (for females of childbearing potential). Participants must also agree to refrain from driving for 8 weeks post CAR-T treatment and be able to reach the site within 2 h in case of emergency.

#### Exclusion criteria

Key exclusion criteria include an inability to provide consent, significant physical or psychiatric conditions that may interfere with the study and known hypersensitivity to the investigational drug or similar agents. Participants with a history of drug, medication, or alcohol abuse, or serious concomitant diseases (e.g., uncontrolled cardiovascular or endocrine conditions) are excluded. Pregnant or breastfeeding individuals, those with active malignancies other than MCL, or infections requiring antimicrobials are not eligible. Other exclusions include chronic HBV infection, HIV, active autoimmune diseases, recent history of thrombosis, severe immunodeficiency, and conditions that may impact safety or efficacy assessments. Additionally, those who received live vaccines within 6 weeks prior to study treatment or have psychological, social, or logistical barriers to participation are excluded.

### Scientific program 

A broad scientific program has been established addressing further exploratory endpoints. At diagnosis, Formalin-Fixed Paraffin-Embedded (FFPE) samples are being reviewed by national reference pathologists and will be assessed again in case of relapse. Central biobanking will be performed, where Peripheral Blood Mononuclear Cells (PBMCs), Dimethyl Sulfoxide (DMSO) stored cells from EDTA tubes, plasma from Streck tubes and cell free DNA will be collected. Molecular remission rates will be measured by next generation sequencing (NGS) from peripheral blood as well as from none marrow at defined time points. From further EDTA samples, immune reconstitution and T cell senescence as well as CAR T cell quantification within the first year after CAR T-cell infusion (Arm A only), are planned. Additionally, plasma proteomics are planned to define plasma-derived exosomes and proteomic signatures as well as protein profiles conferring resistance to BTKi and/ or CAR-T. Quality of life will be assessed by two different questionnaires to be filled in at defined time points pre- and post-treatment, one for physical functioning (assessed with the EORTC QLQ-C30), and one for physical condition/fatigue (assessed with the EORTC QLQ-NHL-HG29). Ultimately, sampling of stool is planned for German patients in order to assess the composition and diversity of the microbiome.

### Statistical methods 

A two-sided stratified log-rank test at a significance level of 10% will be used to compare FFS between Arm A vs. Arm B, testing the null hypothesis (H₀) that there is no difference in FFS between the arms for all time points, against the alternative hypothesis (H₁) that FFS is different between the arms for at least one time point. Analysis will be stratified for country and MIPI risk group (high vs. intermediate/low risk). Based on prior MCL trial data, we assume a median FFS of 27 months for control group B. In this phase 2 trial, sample size was chosen considering feasibility. With 150 patients randomized 1:1, a power of 90% is achieved to reject H₀ with a hazard ratio (HR) of 0.565 (median FFS in Arm A: 48 months), allowing for 10% dropout rate at 5 years. A total of 106 FFS events are required to detect this difference. One interim analysis is planned after 53 events to allow early stop for superiority (O’Brien-Fleming boundaries) or inferiority (Pocock boundaries) of the experimental arm A. The probability to stop early for superiority is 72% with a HR of 0.42 (median 64 months; Arm A) and 32% with a HR of 0.565 (median 48 months). The probability to stop early for inferiority is 63% with a HR of 1.82 (median 15 months) and 22% with a HR of 1.35 (median 20 months). The analysis follows the intention-to-treat (ITT) principle, with intercurrent events such as ineligibility post-registration, non-receipt of investigational medicinal product (IMP), and new lymphoma treatment before treatment failure handled with treatment policy strategy. Initiation of an unplanned new lymphoma therapy in clinical remission constitutes a protocol violation and a hypothetical strategy to handle this intercurrent event will be applied in secondary efficacy analyses. FFS will be described using Kaplan-Meier curves with estimates of FFS probability at yearly intervals. HR with 95% confidence interval and corresponding p value will be calculated from a stratified Cox proportional hazard model. Secondary and exploratory outcomes will be analysed descriptively and exploratively compared between treatment groups.

### Discussion

This is the first international randomized phase II trial investigating the use of CAR T-cell therapy after abbreviated BTKi-based induction in treatment-naïve hr MCL patients. As Ibrutinib may increase T cell fitness, it is integrated into induction therapy prior to T-cell apheresis and will be continued after CAR T-cell infusion as consolidation or maintenance therapy, assuming that it may boost the expansion of CAR T-cells while preventing exhaustion. Chemotherapy is only moderately active in hr disease and can be skipped in responding patients in Arm A after 2 cycles of I + R. This ongoing trial combines highly effective substances with time-limited, intensive treatment, making it a highly innovative and attractive combination. Despite expected heterogeneity within treatment groups, stratified randomization will balance risk profiles between treatment groups to allow reasonable estimation of treatment effects. The broad scientific program with central biobanking will offer valuable information on MRD, immune reconstitution, CAR T-cell quantification and other exploratory items, which will substantially broaden the existing horizon of knowledge about biological features.

As of June 2026, all 5 countries have been activated, and all 43 sites are initiated. An interim analysis is planned when half of the number of events have been observed. Long-term FU is planned within the European MCL Registry, where the disease course in case of relapse and second-line treatments will be documented. When complete, CARMAN will provide important information on efficacy and safety in first-line CAR T cell treatment in high-risk MCL patients and has the potential to prepare a practice changing confirmatory trial for this difficult-to-treat subset of patients.

According to national regulations, the study was approved by the competent authority Federal Institute for Drugs and Medical Devices (BfArM) and received a favorable opinion of the appropriate Ethic’s Committees. The trials is conducted in accordance with Good Clinical Practice guidelines, the German Medicines Act (AMG) and after transition in accordance with EU Regulation 536/2014. The trial is registered on EU Clinical Trial Register: 2022-502405-15-01 for Part I and Part II, submission date 21.06.2023 (ID 9283); substantial modification for Part 1 and Part II submitted on 12.08.2025 (ID 56503).

## Supplementary Information

Supplementary Material 1. SPIRIT checklist.

## Data Availability

No datasets were generated or analysed during the current study.

## Abbreviations

AE(s): Adverse Event(s)
ALL: Acute Lymphoblastic Leukemia
ASCT: Autologous Stem Cell Transplantation
BCL2: B-cell lymphoma 2
BM: Bone Marrow
BR: Bendamustine, Rituximab
Brexu-cel: Brexucaptagene-autoleucel
BTKi: Bruton's Tyrosine Kinase Inhibitor
CAR-T: Chimeric Antigen Receptor T-cell
CD: Cluster of Differentiation
CLL: Chronic Lymphocytic Leukemia
CR: Complete Remission
CRS: Cytokine Release Syndrome
CTLA-4: Cytotoxic T-Lymphocyte-Associated Protein 4
DHAP: Dexamethasone, High-dose Cytarabine, Cisplatin
DMSO: Dimethyl Sulfoxide
ECOG: Eastern Cooperative Oncology Group
EMA: European Medicines Agency
FFS: Failure-Free Survival
FFPE: Formalin-Fixed Paraffin-Embedded
FDA: Food and Drug Administration
FU: Follow-Up
HBV: Hepatitis B Virus
HIV: Human Immunodeficiency Virus
HR: Hazard Ratio / High-Risk (context-dependent)
HRQoL: Health-Related Quality of Life
ICT: Immunochemotherapy
ICE: Immune Effector Cell-associated Encephalopathy (score)
IMP: Investigational Medicinal Product
I+R: Ibrutinib + Rituximab
ITK: IL-2-inducible T-cell kinase
LDH: Lactate Dehydrogenase
LAG-3: Lymphocyte-activation gene 3
Liso-cel: Lisocabtagene maraleucel
MCL: Mantle Cell Lymphoma
MIPI: Mantle Cell Lymphoma International Prognostic Index
MRD: Measurable Residual Disease
NHL: Non-Hodgkin Lymphoma
NGS: Next Generation Sequencing
ORR: Overall Response Rate
OS: Overall Survival
PBMC: Peripheral Blood Mononuclear Cells
PD: Progressive Disease
PD-1: Programmed Cell Death Protein 1
PFS: Progression-Free Survival
PR: Partial Response
QoL: Quality of Life
R-CHOP: Rituximab, Cyclophosphamide, Doxorubicin, Vincristine, Prednisone
R-DHAP: Rituximab, Dexamethasone, High-dose Cytarabine, Cisplatin
SD: Stable Disease
TIM-3: T-cell immunoglobulin and mucin-domain containing-3
Treg: Regulatory T cell
Tisa-cel: Tisagenlecleucel
TP53: Tumor Protein p53
WHO: World Health Organization
VR-CAP: Bortezomib, Rituximab, Cyclophosphamide, Doxorubicin, Prednisone
